# Electron paramagnetic resonance (EPR) spin trapping applied to Chardonnay wines: impact of phenolic content and ethanol

**DOI:** 10.1016/j.crfs.2025.101276

**Published:** 2025-12-11

**Authors:** Pei Han, Alexandre Pons

**Affiliations:** a Univ. Bordeaux, Bordeaux INP, INRAE, OENO, UMR 1366, ISVV, Villenave d’Ornon, F-33140, France; b Bordeaux Sciences Agro, Bordeaux INP, INRAE, OENO, UMR 1366, ISVV, Gradignan, F-33170, France; c Seguin Moreau France, Z.I. Merpins, BP 94, Cognac, 16103, France

**Keywords:** Chardonnay, Wine aging, Fenton reaction, EPR, 1-HER

## Abstract

Chemical oxidation is the main cause of damage during the bottle aging of white wines. It is due to Fenton-type reaction mechanisms, i.e., a radical oxidation cascade which leads to the formation of 1-hydroxyethyl radicals (1-HER) from ethanol. An optimized electron paramagnetic resonance (EPR) approach, using the Fenton reaction and *N-tert*-Butyl-*α*-(4-pyridyl-1-oxide) nitrone as spin trap, was used to study the formation kinetic of 1-HER. Important parameters of the reaction were identified and optimized, i.e., temperature, dissolved oxygen and free bisulfite content. We found that acetaldehyde removed bisulfite without artifacts on 1-HER kinetics, making the protocol suitable for routine laboratory analysis. We then validated the log-normal model to describe the complex formation/degradation kinetic of 1-HER and used it to rank 69 Chardonnay wines in four categories. Low levels of phenolic acid, in particular the cinnamic acids such as caftaric acid, seemed to correspond to a specific formation kinetic of 1-HER (high level produced slowly). In addition, ethanol concentration (0–100 % vol.) greatly impacted its formation kinetics, whereas it had non-significant effect within the white wine range (12–15 % vol.). These results demonstrate that the response of white wines to radical oxidation in controlled and tuned conditions is a promising approach to evaluate aging potential and suggest that maturity level might modulate the 1-HER formation in Chardonnay wines.

## Highlights

• Thermo-accelerated technique to improve EPR analyses in wine.
• Validation of chemical removal of bisulfite for 1-HER analysis by EPR.
• First application of log-normal index on 1-HER kinetic study.
• 1-HER kinetic classification associated with phenolic composition differences.
• No effect of ethanol level of wine (12–15 % vol. range) on EPR signal.

## Graphical abstract

**Figure:**
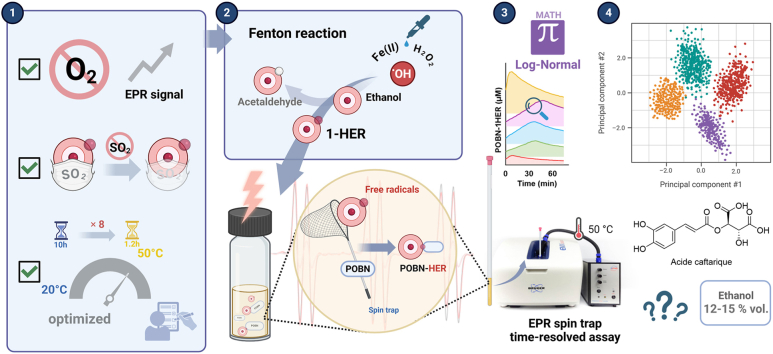

## Abbreviations

EPR: electron paramagnetic resonance
POBN: *N-tert*-Butyl-*α*-(4-pyridyl-1-oxide) nitrone
H_2_O_2_: hydrogen peroxide
OH: hydroxyl radical
1-HER: 1-hydroxyethyl radical

## 1 Introduction

Aging potential is one of the intrinsic qualities of great Chardonnay wines. However, higher oxidative instability results in a loss of aging potential and early oxidation, especially during bottle aging. The oxidative cascade triggered by chemical mechanisms induces the formation of oxidation by-products such as volatile carbonyl compounds (aldehydes or ketones) (Culleré et al., 2007), including methional, phenylacetaldehyde (Escudero et al., 2000) and sotolon (Pons et al., 2008), associated with the loss of aroma quality in young white wines. The composition of the wine, such as pH (Marrufo-Curtido et al., 2022), phenolic compounds (Nikolantonaki et al., 2010) and glutathione content (Pons et al., 2015) as well extrinsic parameters such as temperature (Tarasov et al., 2021) and the quality of the stopper (Pons et al., 2021), are all parameters that can modulate oxidation kinetics during bottle aging.

The relationship between antioxidant or antiradical capacity and the aging potential of wines is now better understood (Waterhouse and Miao, 2021) and has been explored by using Fenton-type oxidation as a model of wine oxidation (Elias et al., 2009a). This chemical mechanism involves the action of transition metals, polyphenols and oxygen (Danilewicz, 2003), which all occur at a wide range of levels in white wine: from 0.69 to 1.71 mg/L of iron (Santos Neto et al., 2019), 0.06–0.42 mg/L of copper (Clark et al., 2015), and trace level to 262–374 mg/L of gallic acid equivalents for phenolic compounds (Landrault et al., 2001). The main phenolic compound families identified and involved in this mechanism are flavan-3-ols, hydroxybenzoic acid derivatives (Waterhouse, 2006) and tyrosol derivatives (Di Tommaso et al., 1998). In addition, molecular oxygen ingress through the closure during bottle aging can be controlled by the winemaker by varying the type and quality of stopper to modulate the shelf life of white wines (Suhas et al., 2024).

Molecular oxygen in the triplet state is weakly reactive due to spin-forbidden reactions, so biomolecules are hardly auto-oxidized by oxygen (Miller et al., 1990). However, in the presence of polyphenols, which are proton suppliers, and transition metals such as iron and copper, oxygen can be reactive and may reduce to form superoxide anion (O_2_^•-^) at the pH level of white wine (pH 3–3.8), the latter occurring in the hydroperoxide radical (·OOH) form Oliveira et al. (2011). This unstable form is reduced to hydroxyl radical (⸱OH). The latter are able to oxide most organic molecules (Buxton et al., 1988).

For this reason, ethanol, the most abundant organic molecule (∼2 M) in wine, is the main substrate to be oxidized and to form hydroxyethyl radicals (HER). These radicals (·OH) preferentially abstract the hydrogen atom from the alpha-carbon of ethanol at a high rate (1.9 × 10^9^ M^−1^s^−1^) to form 1-HER (80–85 %). The formation of 2-HER (1.9 × 10^8^ M^−1^s^−1^) is 10-fold slower (Croft et al., 1992; Pou et al., 1994).

HER and ·OH can be produced by the Fenton reaction at room temperature (Nikolantonaki et al., 2019a; Romanet et al., 2021) or by heating for 10 min after adding Fenton reagent to wine (Marchante et al., 2020a).

Radicals such as 1-HER can be trapped specifically by *N-tert*-Butyl-*α*-(4-pyridyl-1-oxide) nitrone to form the adduct POBN-HER thanks to its stability (Elias et al., 2009a) and the efficacy of the trapping process (Pou et al., 1994; Andersen and Skibsted, 1998), so they can be monitored in real time by EPR spectroscopy. Hence, the formation and degradation kinetic of radicals, especially 1-HER, is of particular interest.

Several studies using the EPR method suggest that endogenous or exogenous compounds having antioxidant properties influence the formation of 1-HER in wine. For example, ascorbic acid (Marchante et al., 2020a) and bisulfite (Elias et al., 2009a) had a strong inhibitory effect on 1-HER formation in a concentration-dependent manner. The elimination of bisulfites in wines before EPR analysis is thus crucial for revealing the intrinsic formation capacity of free radicals like 1-HER in wine. Bisulfites can be eliminated by the stepwise addition of peroxide hydrogen (Elias et al., 2009a) or CO_2_ bubbling (Nikolantonaki et al., 2019a).

There are two main methods for applying EPR to wine. The first evaluates the level of free radicals at a specific time after forced oxidation (Elias et al., 2009b; Kreitman et al., 2013), whereas the second is kinetic-based and focuses on the evolution of the signal intensity (or concentration) of a specific radical over time (Nikolantonaki et al., 2019a). This requires characterizing the kinetic curve of the radical as it evolves (Marchante et al., 2020b; Nikolantonaki et al., 2019b).

Although the spin trap/EPR method has previously been used to characterize and quantify free radicals in wine, it has not yet been fully validated. Moreover, the effects of several key operational parameters of the Fenton/POBN reaction (such as oxygen, temperature or bisulfite) have not been systematically examined. So, based on previous work carried out by Elias and Waterhouse (2010) and Nikolantonaki et al. (2019a), this manuscript proposes to contribute to the improvement of our knowledge on the application of Spin Trap/EPR methodology on white wines to determine their oxidative susceptibility. This work describes a new optimization and first analytical validation of Spin Trap/EPR methodology based on a log-normal model proxy. We used the method to classify a wide range of Chardonnay wines from the Burgundy region and explore the relationship with their composition (mainly ethanol and phenolic compounds).

## 2 Materials and methods

### 2.1 Chemicals

All chemicals were of analytical grade. The water used was purified by a Milli-Q system (Millipore, Saint-Quentin-en-Yvelines, France). Hydrogen peroxide (30 % vol.), iron (II) sulfate heptahydrate (≥99 %), acetaldehyde (≥99.5 %), *L* (+)*-*tartaric acid (≥99.7 %) and sodium hydroxide (≥98 %), gallic acid (≥99 %), caffeic acid (≥98 %), tyrosol (≥98 %), *trans*-resveratrol (≥99 %), *p*-coumaric acid (≥98 %), vanillic acid (97 %), caftaric acid (≥97 %), 3-hydroxytyrosol (≥98 %) and trifluoroacetic acid (99 %) were purchased from Sigma-Aldrich (St. Louis, USA). (+)-Catechin monohydrate (≥98 %) was purchased from ChemCruz (Heidelberg, Germany). The spin trap *α*-(4-pyridyl-1-oxide)-*N*-*tert*-butylnitrone (POBN, 98 %) and (−)-epicatechin (≥97 %) was obtained from TCI (Zwijndrecht, Belgium). Ferulic acid (≥99 %), ethanol absolute (>99.7 %, VWR, Pennsylvania, USA), hydrochloric acid (37 %) and acetonitrile (≥99.9 %) was purchased from Fisher Scientific (Illkirch, France).

### 2.2 Wine samples

This study was conducted with commercial Chardonnay wines selected and provided by the Bourgogne Wine Board (BIVB), with a subset of wines (Table 1) selected to optimize and validate the EPR methodology. A further set of wines (N = 69) covering the 1997 to 2022 vintages from different appellations (Table S3) was analyzed. Wines were produced according to local winemaking protocols including alcoholic and malolactic fermentations and, according to the winery, with an additional maturation period in oak barrels. No ascorbic acid was added at bottling. The number of replicates is indicated, capital letters for biological replicate (N), and lowercase letters for analytical replicate (n). All the wines were sealed with natural or microagglomerate corks. Before analysis, all samples were stored at a constant temperature (10 °C) at the BIVB facility.

**Table 1:** Origin and composition of wines for evaluation of different parameters on EPR signal as well as for analytical validation of 1-HER quantitation.

Wines	Appellation	Vintage	OD_420_^a^	Alc. b	TA c	pH	Tar. d	SO_2_^e^	Test f
W1	Côte de Nuits	2022	0.09	13.4	4.1	3.3	3.0	<3	T
W2	Côte de Nuits	2022	0.06	12.0	5.2	3.1	3.5	<3	T
W3	Côte Chalonnaise	2019	0.13	14.3	4.8	3.3	4.0	8.3	T
W4	Bourgogne	2021	0.03	12.6	3.7	3.4	3.2	13	T, SO_2_
W5	Côte de Beaune	2020	0.05	13.3	4.4	3.2	4.3	12	T
W6	Côte Chalonnaise	2019	0.11	14.0	4.3	3.3	4.1	4	T, SO_2_
W7	Côte Chalonnaise	2019	0.18	13.2	4.2	3.3	3.5	5	DO, SO_2_, R
W8	Côte de Nuits	2022	0.09	11.0	5.3	3.0	3.4	14.2	EtOH
W9	Bourgogne	2017	0.15	12.0	3.5	3.4	3.0	<3	EtOH
W10	Mâcon	2020	0.11	12.9	3.0	3.5	3.2	30	SO_2_
W11	Mâcon	2020	0.14	12.9	3.0	3.5	3.2	24	R
W12	Côte de Beaune	2020	0.13	13.0	3.9	3.3	3.4	24.5	R

a Optical density at 420 nm.

b Ethanol (% vol.).

c Total acidity (H_2_SO_4_ g/L).

d Tartaric acid (g/L).

e Free bisulfites (mg/L).

f Application of different tests or analytical procedures on 1-HER formation kinetic according to wine sample: T: impact of temperature, DO: impact of dissolved oxygen, SO_2_: optimization of method to remove free bisulfites, R: tests for repeatability and reproducibility validation, EtOH: impact of ethanol.

### 2.3 Enological parameters

Dissolved oxygen (DO) was measured by NOMASenseP6000 with Pst3 sensors (PreSens, Regensburg, Germany). The free sulfur dioxide levels of these wines were measured by a digital wine analyzer (Sentia™, Australia) with test strips, and the enological parameters (ethanol, pH, total acidity, tartaric acid) were measured by Foss WineScan 79000 FTIR (FOSS, Nanterre, France). Optical density at 420 nm (OD_420_) was evaluated by UV–Vis spectrophotometer V-730 UV–Vis (JASCO, Lisses, France).

### 2.4 EPR spin trapping method

#### 2.4.1 Preparation of sample

Fifty μL of acetaldehyde solution (567.5 mM in ethanol) were added to 10 mL of a white wine sample and incubated at 4 °C for 12 h to bind and remove free bisulfites. The pretreated samples were then stored at 4 °C for up to an additional 12 h before analysis. The storage period (12–24 h post-addition at 4 °C) had no significant impact on the EPR signal after initiation of the Fenton reaction. The DO of samples was desorbed by bubbling with pure nitrogen gas (<0.1 mg/L) before each analysis.

#### 2.4.2 Initiation of radical oxidation

The forced oxidation protocol was based on previous studies (Elias and Waterhouse, 2010; Nikolantonaki et al., 2019a) and adapted as follows. POBN (5.8 mg) was dissolved in 1 mL pretreated wine sample followed by sequential addition of 5 μL of FeSO_4_ solution (10 mM) and 5 μL of H_2_O_2_ solution (120 mM) to trigger Fenton oxidation. The reagent solutions were prepared every day before EPR experiments. The mixture was gently vortexed before loading into a 50 μL clear disposable microcapillary tube (BRAND, Schnelldorf, Germany) and was then sealed with wax (Hirschmann-Laborgeräte, Eberstadt, Germany). The microcapillary tube containing the reactional mixture was inserted into the EPR cavity and analyzed by a EPR EMXnano spectrometer (Bruker BioSpin, Rheinstetten, Germany).

#### 2.4.3 EPR analysis

EPR analysis was carried out in 2D field delay acquisition mode and spectra were acquired 2 min after triggering of the Fenton reaction. Recording took place every 120,000 ms with three scans. In the first experiment, the effect of temperature was assessed between 20 and 50 °C and was controlled by a constant air flow in the resonance cavity, without any contact with wine samples (Noxygen, Elzach, Germany). The optimal temperature was set at 50 °C for the remaining experiments. The instrument parameters were set as follows: center field 3426.25 G, sweep width 60 G, microwave power 10 mW, modulation amplitude 1 G, modulation frequency 100 kHz, sweep time 4 s, receiver gain 40 dB, power attenuation 25 dB. EPR instrument control and data processing were performed with Xenon Nano software (version.1.3, Bruker Biospin). Simulation and quantitation of 1-HER were conducted using the Spinfit and Spincount tools, respectively. POBN-HER simulation spectra were from the spectral library.

### 2.5 Optimization of EPR method

#### 2.5.1 Impact of dissolved oxygen

The effect of dissolved oxygen (DO) on the EPR signal was evaluated in both synthetic (pH 3.5, 12 % vol., 5 g/L tartaric acid) and commercial wine (W7, Table 1). Six levels were selected, 0.1, 3, 6, 12, 20 mg/L, and adjusted by bubbling pure oxygen (99.99 %, Messer, France) or nitrogen at room temperature. Radical oxidation was initiated by Fenton reaction as described and the kinetic of 1-HER formation was monitored in triplicate at 50 °C by Spin Trap/EPR.

#### 2.5.2 Acetaldehyde bisulfite binding test

Tests were carried out on a wine (W7, Table 1) initially with a low bisulfite level (5 mg/L), adjusted to 30 mg/L by adding sodium metabisulfite. Sample was kept in the dark for about 12 h before use. Then, 50 μL acetaldehyde (567.5 mM, EtOH) were added to 10 mL of wine and five incubation times were tested: 10 min, 1 h, 3 h, 12 h, and 24 h at 4 °C. Thereafter, the protocol was similar to that previously described, i.e., DO was desorbed with pure nitrogen before analysis of radicals by EPR. Tests were carried out in triplicate.

Following the determination of the optimal incubation time with acetaldehyde, three wines with different bisulfite levels were tested: high (30 mg/L, W10), medium (13 mg/L, W4) and low (4 mg/L, W6).

#### 2.5.3 Method validation tests

The repeatability of the method was evaluated with three Chardonnay wines (Table 1) from the 2019 and 2020 vintages, with different initial bisulfite concentrations: W7, W11, W12; 5, 24, 24.5 mg/L respectively. The wines were tested nine times on the same day to assess intraday repeatability using the optimized EPR method, as previously described. The reproducibility of the method was evaluated using the same Chardonnay wine (W7) over a six-day period. All reagents were freshly prepared, whereas the wine sample was stored in an inert container at 4 °C to prevent oxidation.

### 2.6 Impact of ethanol

The impact of ethanol on the formation of 1-HER was evaluated in both model solution and wines. The composition of the model solutions prepared daily was 5 g/L tartaric acid, pH 3.5 using Milli-Q water with 14 levels of ethanol concentration: 0 %, 1 %, 5 %, 10 %, 12 %, 13 %, 14 %, 15 %, 16 %, 20 %, 40 %, 60 %, 80 %, and 100 % vol. Three wines were also selected based on their low/medium ethanol level (Table 1): W8 (base wine, 11 % vol.) and W9 (12 % vol.). The effect of ethanol was evaluated by adding increasing quantities of ethanol: 12 %, 13 %, 14 %, and 15 % vol. to cover the range found in commercial Chardonnay wines. Following a 12-hr incubation period, the samples underwent analysis using the optimized EPR method, as detailed in the previous section. All tests were conducted in triplicate.

### 2.7 Analysis of phenolic compounds

Phenolic compounds were quantified with a previously described method (Ćurko et al., 2014; Gürbüz et al., 2007), but with modifications as described below. Analyses were carried out using high-performance liquid chromatography (HPLC, Thermo Scientific, MA, USA) consisting of a Vanquish autosampler and a Vanquish pump equipped with an online degasser, coupled to a fluorescence detector (UltiMate 3000, Dionex). Chromatographic separation was carried out on an OSDII C18 column (5 μm, 250 × 4.6 mm; Thermo Scientific, Waltham, MA, USA). The mobile phases consisted of solvent A (Milli-Q water containing 0.5 % trifluoroacetic acid, TFA) and solvent B (65 % aqueous acetonitrile with 0.5 % TFA). Elution was performed using a stepwise gradient at a flow rate of 1.0 mL/min as follows: 8 % of B for 8 min, 8–15 % of B over 4 min, 15 % of B for 3 min, 15–20 % of B over 16 min, 20–100 % of B over 2 min, 100 % of B for 10 min, followed by re-equilibration at 8 % B for 3 min. Wine samples were filtered (through 0.2 μm PTFE syringe filters) prior to injection (5 μL). The excitation (λ ex) and emission (λ em) wavelengths used to quantify the phenolic compounds were optimized and are listed in Table S5.

### 2.8 Statistical analysis and data processing

#### 2.8.1 Statistical analysis

Analysis of variance (ANOVA) or the Kruskal-Wallis test was applied depending on normality and homoscedasticity of replicate analysis. Significant differences between groups were considered with a p value < 0.05. If necessary, a log transformation was applied in order to validate the normality and homoscedasticity tests. Pairwise comparison was performed by Student's *t*-test and multiple pairwise comparisons were performed by the Tukey or Dunn test. Principal component analysis (PCA) and the k-Means clustering algorithm (KM) were performed by XLSTAT software (version 2023.3.1). KM used the Euclidean distance and determinant (W) as the clustering criterion. The optimal number of clusters was determined by the elbow method.

#### 2.8.2 Log-normal fitness

In our experimental conditions, the kinetic curves of 1-HER formation adducts during the reaction in white wines followed a log-normal fit. The data were fitted by Sigma plot software (CA, USA) from the probability density function of log-normal to obtain four parameters for each wine sample. According to Limpert et al. (2001), the log-normal distribution can be characterized by the mean (μ) and the standard deviation (σ). The anti-logarithm of μ gives the geometric mean (*index x*_*0*_) = e^μ^, which is widely considered to be the most intuitive parameter to describe the variable in skewed data. The standard mathematical equation (**Eq. A**) of log-normal was modified and adapted as follows:

Eq. (A) $$F\left(x\right)=y_{0}+\left(a/x\right)\;\exp\left(-0.5\;{\left(\ln\left(x/x_{0}\right)/b\right)}^{2}\right)$$

$Index\;a:a=AUC/b\sqrt{2\pi }$

The area under the curve (AUC) was calculated based on the corrected baseline, *index a* is related to the AUC, amplitude and *index b* value. The *index a* allows the overall form of the kinetic curve to be determined.

**Fig. 1:**
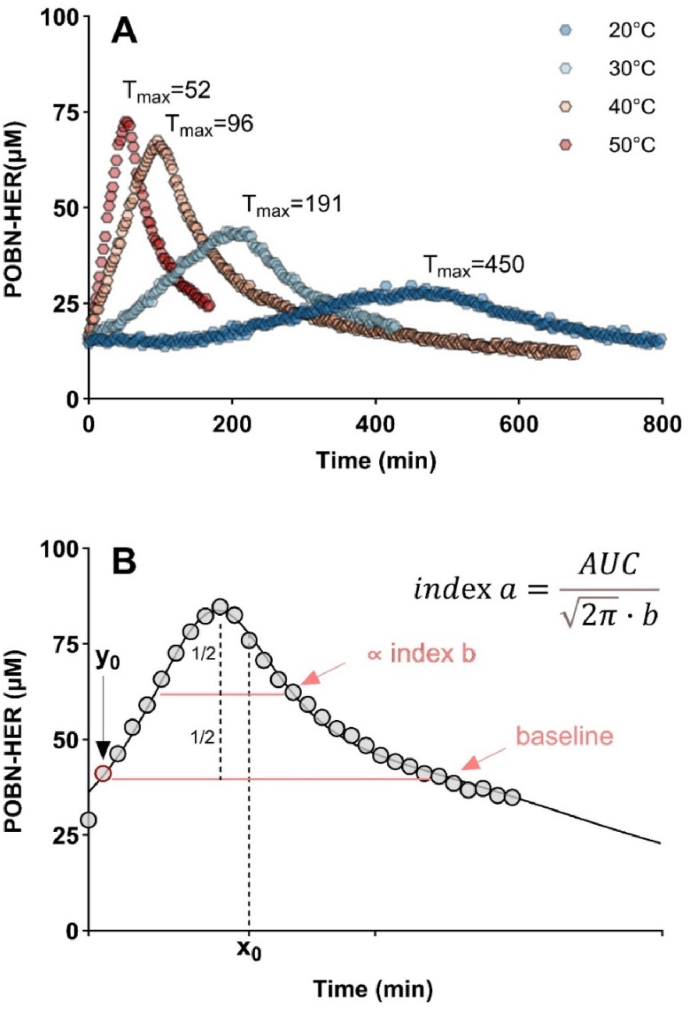
(**A**) Impact of EPR cavity temperature (20–50 °C range) on 1-HER formation kinetic in wine W1. T_max_: time to reach peak of curve (min). (**B**) Schematic representation of kinetic formation of POBN-HER at 50 °C. Parameters *x*_*0*_, *y*_*0*_, *a* and *b* correspond to those of the log-normal function fit (**Eq. A**).

Indexb:b=σ(standarddeviation), is proportional to the full width at half maximum (FWHM) of the corrected baseline (Fig. 1B).

$Index\;x_{0}:{x_{0}=e}^{\mu }\;\left(Geomean\right)\text{.}$ *Index x*_*0*_ is a location parameter that represents the time needed to reach the theoretical median value of the concentration of radicals.

$Index\;y_{0}\text{:}$ Shift of first fitting point on y axis to determine the corrected baseline.

## 3 Results and discussion

### 3.1 Characterization of POBN-HER formation kinetic

#### 3.1.1 Identification of POBN-HER adduct

The formation of radicals was initiated by Fenton reaction*,* i.e., iron (II) and hydrogen peroxide (H_2_O_2_) produce hydroxyl radicals (·OH), thereby inducing ethanol oxidation and 1-HER intermediates (Elias et al., 2009b). Spin trap POBN was used to trap 1-HER. The first step of our work confirmed the formation of the typical six-line EPR spectra specific to POBN-HER adducts. The hyperfine coupling constants were a^N^ = 15.47 G and a^H^ = 2.56 G, which correspond to previous reports (Buettner, 1987; Elias et al., 2009a; Nikolantonaki et al., 2019a). The adduct was fitted with POBN-1HER simulation spectra with a g factor 2.006, line shape 0.8635 and line width 1.101 G.

#### 3.1.2 Optimization of reaction temperature

As in previous EPR studies on wines (Elias and Waterhouse, 2010; Nikolantonaki et al., 2019a), our preliminary studies were carried out at room temperature. We observed a quasi-linear low kinetic accumulation of POBN-HER adducts within the first hour, as already observed (Nikolantonaki et al., 2019a; Romanet et al., 2021). Preliminary investigations showed that some white wines had similar evolution trends during the first hour of kinetics but then demonstrated specific trends after 2 h or more (Fig. S1). We therefore extended the analysis time to take advantage of the overall kinetic curve of the radical formation or degradation to 4 h, 6 h and 10 h. The best results were obtained by monitoring the reaction for 10 h. However, this is not feasible for the routine analysis of many wine samples, so we investigated the effect of the resonance cavity temperature (20 °C–50 °C) on the kinetics of radical formation in six white wines (Table 1). We ensured that the rise in temperature did not modify the overall trend of radical production.

Fig. 1A shows the impact of temperature on the EPR signal. When the measurement was carried out at room temperature (20 °C), T_max_ was reached after 450 min, whereas it took almost 52 min at 50 °C, i.e., nearly 10 times less. The method increased the signal intensity of free radicals (higher amplitude of triplet peak), and it improved the discrimination level between the different wines (Table S1). To our knowledge, this is the first demonstration of the strong impact of cavity temperature on the formation of free radicals in white wines.

In our experimental conditions, the concentration of the POBN-HER adduct increased over time to reach a peak (increasing phase, Fig. 1B). It then decreased as the reaction time increased (decreasing phase), as already reported (Nikolantonaki and Waterhouse, 2012). To our knowledge, the overall shape of the curve (in both phases) has never been considered when evaluating the production of POBN adducts. Two hypotheses might explain this decline. Firstly, dimerization of POBN-HER has already been reported to produce diamagnetic compounds, resulting in a decreasing trend of POBN-HER after reaching a peak (Nikolantonaki and Waterhouse, 2012). Therefore, this declining phase could provide information about the total amount of radicals in the matrix. Another possibility is that nitroxide adducts POBN-HER may be reduced to silent hydroxylamine in the presence of a reductant such as glutathione (GSH), a phenomenon already observed in biological research (Stoyanovsky and Cederbaum, 1998).

#### 3.1.3 Characterization of kinetic curve

Previous methods for characterizing the kinetic profile of free radicals in alcoholic beverages included the calculation of lag time (Uchida et al., 1996) and the area under the curve (AUC) in beer studies (Porcu et al., 2022). Concerning enological research, the parameters include the initial formation rate (slope), the maximum concentration ([max]) of formed radicals (Nikolantonaki et al., 2019a, 2019b; Romanet et al., 2021) and the time to reach the maximum concentration (T_max_) or the initial concentration of radicals (T_0_) (Márquez et al., 2019). Each research team concentrated on a single part of the kinetic curve for various analytical reasons.

Based on our experimental conditions, the kinetic of radical evolution during the reaction follows a log-normal function with a goodness of fit of 96–97 % (Table S2). Based on Limpert et al. (2001), four parameters called *y*_*0*_, *x*_*0*_, *b* and *a* describe this function (Fig. 1B) and account for the shape of the curves, including their height and width as well as scaling and shifts in peak.

We evaluated their ability to delineate the kinetic curve and discriminate between different curves obtained from different wine samples. In this regard, *a* and *x*_*0*_ were of particular interest for further investigation (Fig. S2).

### 3.2 Optimization of the method

#### 3.2.1 Impact of dissolved oxygen

**Fig. 2:**
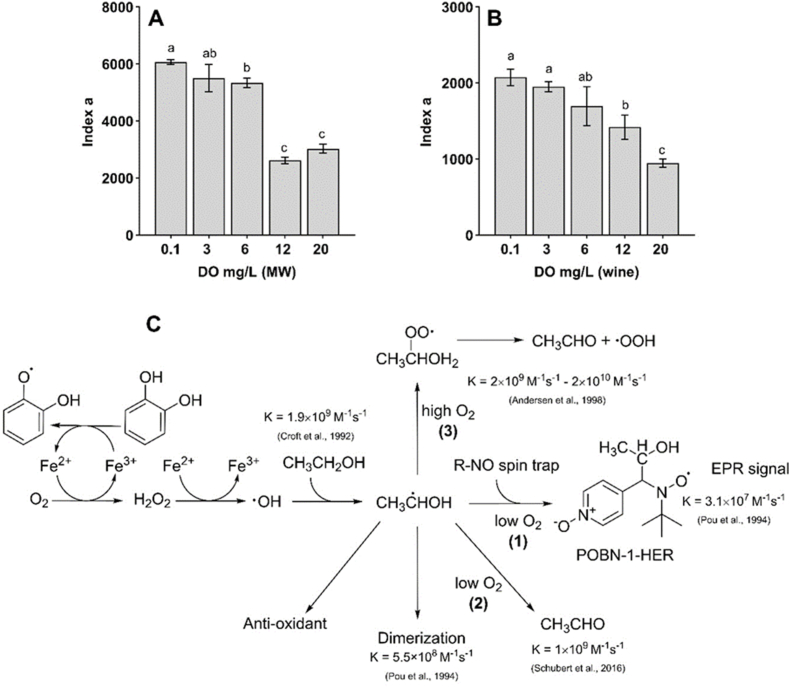
Impact of dissolved oxygen (DO) concentration in model wine (MW, **A**) and Chardonnay wine W7 (**B**) on the *index a* value calculated from the POBN-HER formation kinetic log-normal model (n = 3, Tukey test, p < 0.05). (**C**) Chemical formation/evolution pathway of 1-HER during white wine oxidation based on previous work (Elias and Waterhouse, 2010).

DO in both synthetic and commercial wines was adjusted at five levels from 0.1 to 20 mg/L (Fig. 2A and B) before analysis by EPR. Three and six mg/L correspond respectively to the average DO levels usually detected in wines in laboratory conditions.

Results showed that the DO level of white wines significantly affects the kinetic of radical evolution during the reaction (Fig. 2B). High dissolved levels of oxygen led to a significant decrease in *index a,* corresponding to a lower and slower POBN-HER formation kinetic.

This phenomenon was previously observed in beer where a high oxygen content produced more acetaldehydes through 1-HER via 1-hydroxyethyl superoxide radical intermediates (Andersen and Skibsted, 1998). It may be explained by the rate constant (K) of the three reactions involved in this process (Fig. 2C). In fact, 1-HER can react with oxygen at rates ranging from K = 2 × 10^9^ M^−1^s^−1^ (Pou et al., 1994) to K = 2 × 10^10^ M^−1^s^−1^ (Andersen and Skibsted, 1998) (**pathway 3**). According to Pou et al. (1994), this reaction is much faster than the trapping reaction of 1-HER by POBN (K = 3.1 × 10^7^ M^−1^s^−1^, **pathway 1)**. In addition, according to Schubert et al. (2016), 1-HER could very quickly lead to acetaldehyde (K = 1 × 10^9^ M^−1^s^−1^, **pathway 2**) or other products (acetic acid, 2,3-butanediol by self-recombination). Conversely, this result indicates that deoxygenation of samples prior to EPR analysis is useful for increasing the EPR signal of 1-HER by about 6–13 %.

#### 3.2.2 Impact of free bisulfites and their removal

**Fig. 3:**
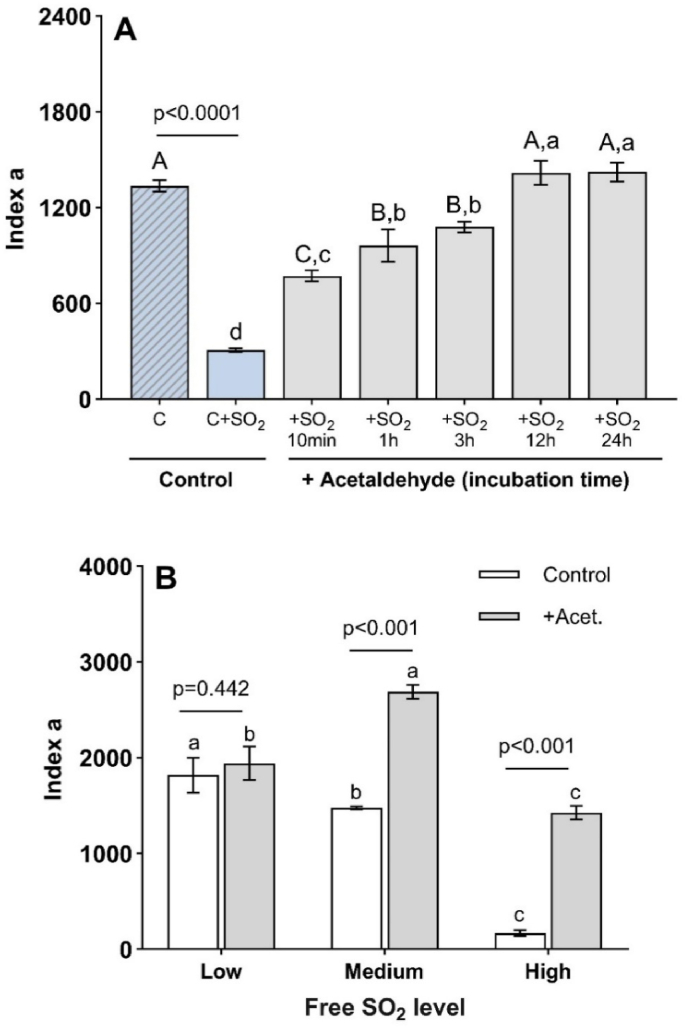
(**A**) Impact of incubation time with acetaldehyde (2.8 mM) on the recovery of the EPR signal (*index a*) in a young commercial white wine with low bisulfite content (C, striped blue bar) spiked with bisulfites (C + SO_2_, 30 mg/L, blue bar), p value indicates level of significance between them (n = 3, Student t-test, p < 0.05). Uppercase letters correspond to significant difference between the control wine (C) and treatments (10 min–24 h of incubation) whereas lowercase letters correspond to significant difference between incubation time with acetaldehyde and C + SO_2_ modality (n = 3, Tukey test, p < 0.05). (**B**) Impact of acetaldehyde addition on *index a* value of three white wines with different bisulfite levels: high (30 mg/L, wine W10), medium (13 mg/L, wine W4) and low (4 mg/L, wine W6). P values indicate level of significance between treatments for each wine (n = 3, Student t-test, p < 0.05). Different letters indicate significant differences between wines for each treatment (n = 3, Tukey test, p < 0.05).

Bisulfites are traditionally and widely used in winemaking and especially before bottling to confer protection against oxidation during bottle aging. The impact of bisulfites on the inhibition of the formation of 1-HER in white wines (Nikolantonaki et al., 2019a) has already been demonstrated. This effect is linked with its reaction with hydrogen peroxide (Danilewicz et al., 2008), hydroxyl radical (⸱OH) and 1-HER. In our experimental conditions, we also observed that bisulfites impacted the formation of POBN-HER in a dose-dependent manner: the white wine containing bisulfites produced very low radical levels when the Fenton reaction was triggered (Fig. 3A). Hence, bisulfites mask the real capacity of a wine to produce radicals. Therefore, the removal of bisulfites is essential when assessing an enological potential to generate free radicals. This can be achieved through various methods, including the controlled addition of hydrogen peroxide (Elias et al., 2009a) and bubbling with CO_2_ (Nikolantonaki et al., 2019a).

To adapt our analytical method and improve the effectiveness of bisulfite removal, we developed an optimized chemical approach to remove free bisulfites based on their high reactivity with acetaldehyde (Burroughs and Sparks, 1973; Coetzee et al., 2018). Tests were carried out on several wines (Table 1) containing different initial bisulfite levels to evaluate the impact of acetaldehyde addition on the EPR signal.

The results confirmed that, under our experimental conditions, the addition of bisulfite to a wine inhibits the 1-HER signal. While binding free bisulfites, the addition of acetaldehyde results in a dose-dependent increase in 1-HER signal intensity (Fig. 3B). Incubation with acetaldehyde for 12 h allowed the EPR signals to return to normal and remain stable for 24 h.

We applied this protocol to three wines from different vintages and appellations (Table 1), with high, medium and low levels of bisulfite. Each wine presented significant differences in its *index a* value according to the level of bisulfite in the wine: Low > Medium ≫ High. After the treatment, the results between the wines were still significant but the ranking was completely different (Medium > Low > High), with “High” modality showing the highest increase (factor eight). These results suggest the unique intrinsic composition of wine “Medium” corresponding to the highest formation of radicals (highest *index a*).

The addition of acetaldehyde produced this ‘revelation’ effect, whereas the wine with low free bisulfites exhibited the same EPR signal after the treatment (Fig. 3B), i.e., it did not induce artifactual values of *index a*.

### 3.3 Validation of method

The intraday repeatability of three Chardonnay wines (Table 1) from different appellations was examined. Each wine was analyzed nine times using the optimized EPR method within the same day. The reproducibility of the method was evaluated using the same Chardonnay wine over a six-day period. We selected eight parameters describing the curve of the formation kinetic of 1-HER. The performance of the analytical method was expressed in terms of relative standard deviation (RSD). The repeatability and reproducibility of all EPR parameters ranged between 2 % and 8 %, and between 2 % and 6 % according to the analytical parameter from the fit of log-normal (Table S2), enabling its application in white wines.

### 3.4 Evaluation of formation kinetic of 1-HER in Chardonnay wines

**Fig. 4:**
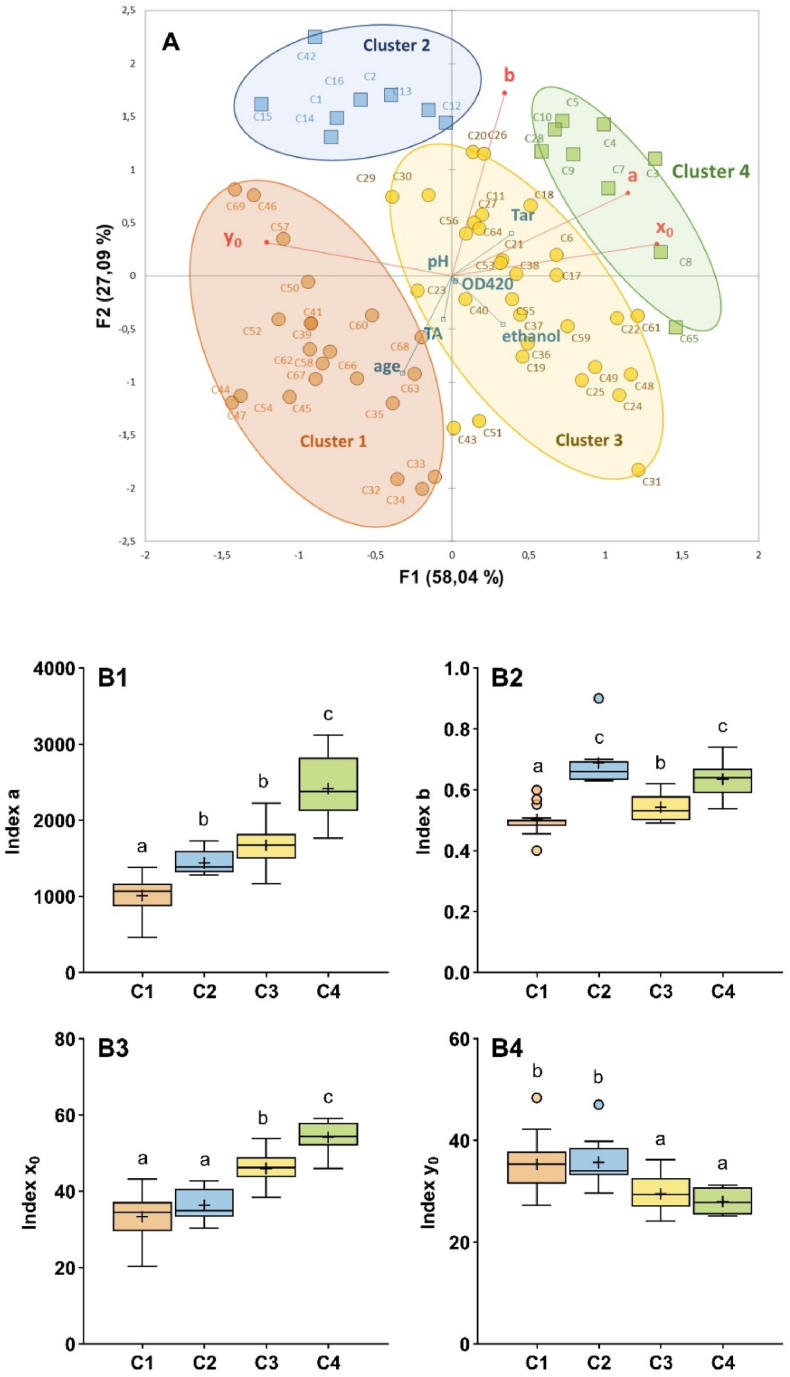
(**A**) Principal component analysis of EPR signatures (*a*, *b*, *x*_*0*_ and *y*_*0*_) of 69 young and old Chardonnay wines. Active variables are in red and supplementary variables in blue. The four colored ellipses correspond to k-means clustering (C): C1 (N = 22, in orange), C2 (N = 8, in blue), C3 (N = 30, in yellow) and C4 (N = 9, in green). (**B**) Box plot of the four *indexes* according to k-means clustering: *index a* (**B1**), *index b* (**B2**), *index x*_*0*_ (**B3**), *index y*_*0*_ (**B4**). Different letters correspond to significant differences between clusters (Kruskal-Wallis, Dunn post-hoc test, p < 0.05). Mean values are indicated by “+“.

To investigate differences in 1-HER radical formation kinetics among Chardonnay wines, the optimized method was applied to 69 wines from Burgundy, spanning different vintages and a wide range of composition (Table S3): ethanol (11.9–14.2 % vol.), pH (3.1–3.5) or DO_420_ values (0.01–0.43). Principal component analysis (PCA) was applied to the overall data set (Fig. 4A). The first two principal components (F1 and F2) accounted for 85.13 % of the total variance. Axis F1 explaining 58.04 % of the total variance was primarily driven by EPR parameters such as *index a*, *x*_0_ and *y*_*0*_. In contrast, axis F2, accounting for 27.09 % of the variance, was mainly associated with *index b*.

Distribution of wines in the PCA representation was quite complex and was improved by unsupervised clustering according to their kinetic characteristics. The K-Means clustering algorithm was applied by minimizing the within-cluster variance, representing the variance of samples around their respective centroids (Jain, 2010). As a result, four distinct clusters were identified (Fig. 4A), each showing significant differences in the values of the log-normal model parameters (Fig. 4B). The largest differences were obtained between clusters 1 and 4 for the four *indexes*, especially with *index a* (factor 6 between the lowest and highest values).

Cluster 1 (orange ellipse) included wines with the largest range of vintages from 1997 to 2022, showing a rapid radical production at the beginning (higher *y*_*0*_, ${\bar{y}}_{0}$ = 35.3), with the peak occurring early in the reaction (lower *index x*_*0*_, ${\bar{x}}_{0}$ = 33.4) whereas the overall radical production was relatively low (corresponding to lower values of both *index a* and *b;* $\bar{a}$ = 1011, $\bar{b}$ = 0.50). Comparatively, cluster 4 (green ellipse) corresponds mainly to younger wines from the 2014 to 2022 vintages with the highest average *index a* value ($\bar{a}$ = 2419) accompanied by a shift in geometrical means *index x*_*0*_ (${\bar{x}}_{0}$ = 54.3). These two indexes indicate slower formation kinetics accompanied by a large amount of 1-HER radical production during the reaction (Fig. S3).

In parallel, we studied the distribution of the enological parameters according to the four different clusters (Table S4). The ethanol level was significantly lower in cluster 2 (p = 0.034), so we hypothesized that variations in ethanol content might be linked to slight differences in EPR kinetic parameters. Additionally, given the potential antioxidant or prooxidant properties of phenolic compounds, we further assessed their distributions.

### 3.5 Distribution of phenolic compounds based on 1-HER kinetic parameters

Eleven main phenolic compounds were quantified including tyrosol, hydroxytyrosol, vanillic acid, ferulic acid, caffeic acid, caftaric acid, *p*-coumaric acid, gallic acid, catechin, epicatechin, and *trans*-resveratrol (Table S6). Most have a varietal origin, apart from tyrosol and hydroxytyrosol which are produced during alcoholic fermentation (Álvarez-Fernández et al., 2018), and gallic acid which is released during oak wood maturation (Alañón et al., 2011) or via the hydrolysis of hydrolysable tannins (Waterhouse, 2006).

Based on spectrophotometric assays, the antioxidant capacity of wines is generally attributed to their phenolic content and reactivity with oxygen and reactive oxygen species (de Quirós et al., 2009; Landrault et al., 2001; Makris et al., 2003), mainly due to the hydroxyl and methoxy groups on the aromatic ring (Danilewicz, 2007) as well as unsaturated side chains (Gislason et al., 2011). For example, compounds such as catechin not only modulate oxygen consumption (Escudero et al., 2025), but also contribute to the formation of ortho-quinones, the latter of which is directly involved in oxidation processes (Oliveira et al., 2017). Additionally, caftaric acid and esterified cinnamate are related to the high antiradical capacity of white wines (Makris et al., 2003). Stilbenes such as *trans*-resveratrol have also been identified in white wines, although usually only in trace amounts (Gürbüz et al., 2007).

According to the EPR kinetic parameters and phenolic composition, Spearman correlation coefficient values were low but significant (Table S7). For example, *indexes a*, *b*, and *x*_*0*_ showed negative correlations with the total phenolic content, with *index a* exhibiting the highest correlation (r = - 0.357, p = 0.003), suggesting that the initial phase of radical formation could be influenced by phenolic compounds.

**Fig. 5:**
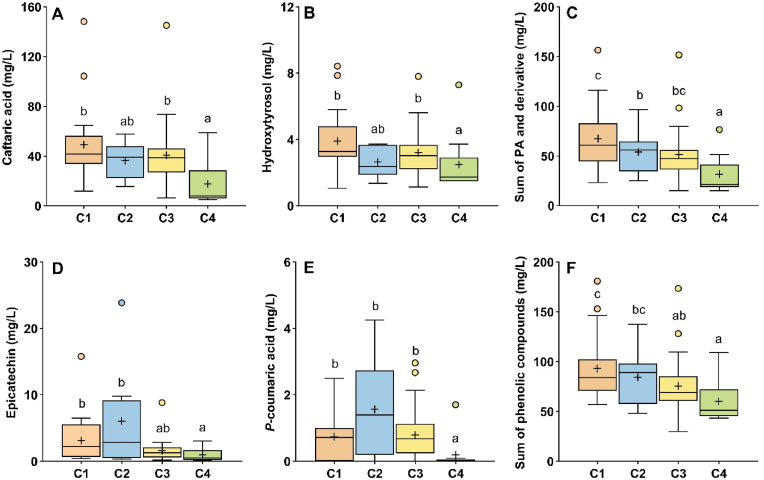
Concentration of molecular phenolic compounds in four Chardonnay wine clusters classified by EPR indexes. Mean values are indicated by “+“. Analytes include caftaric acid (**A**), hydroxytyrosol (**B**), the sum of assayed phenolic acids and derivative (**C**), epicatechin (**D**), *p*-coumaric acid (**E**), and the sum of all assayed phenolic compounds (**F**). Different lowercase letters indicate statistically significant differences between the four clusters (Kruskal-Wallis, Dunn post-hoc test, p < 0.05).

Based on clustering results (Fig. 4), the highest *index* $\bar{a}$ value was found in Cluster 4 along with lower concentrations of several phenolic compounds (Fig. 5), including caftaric acid, *p*-coumaric acid, epicatechin, and hydroxytyrosol. Based on our sample selection, we also observed a low but significant correlation between log normal indexes and phenols (Table S7), suggesting a potential link between these compounds and the formation kinetic of 1-HER, as previously observed by Nikolantonaki et al. (2019a).

In addition, caftaric acid and *p*-coumaric acid are of particular interest as they are known to play key roles in white wine oxidation (Kallithraka et al., 2009). Despite the relatively broad concentration range of phenolic compounds, their limited impact on radical kinetic parameters suggests the involvement of additional contributing species.

**Fig. 6:**
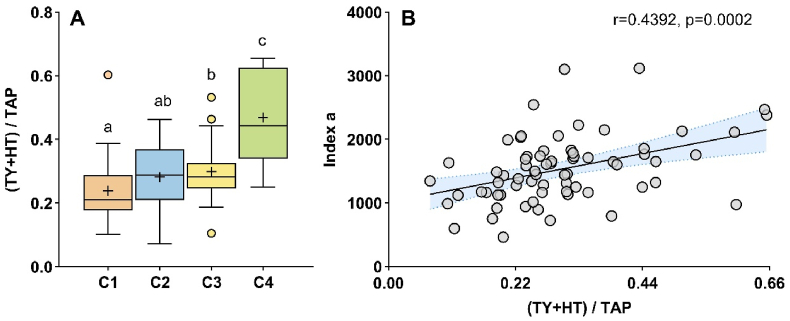
Box plot representation of the ratio of tyrosol (TY) and hydroxytyrosol (HT) to total assayed phenolics (TAP) in four Chardonnay wine clusters (**A**) and their correlation with *index a* (**B**). Mean values are indicated by “+“. Different letters indicate statistically significant differences among clusters (Kruskal-Wallis test and Dunn's post hoc test, p < 0.05). In **B**, correlations were assessed using Spearman test (n = 69); trend lines indicate the direction of correlation and blue shaded areas represent the 95 % confidence intervals.

Given the complexity of phenolic composition and their antagonist distributions, we assessed the proportion of phenolic compounds. As illustrated in Fig. 6, the proportion of tyrosol and its derivative hydroxytyrosol (both derived from yeast metabolism) relative to the total of the eleven assayed phenolics showed a significant positive correlation with *index a* (r = 0.44, p < 0.05).

### 3.6 Impact of ethanol on 1-HER formation

**Fig. 7:**
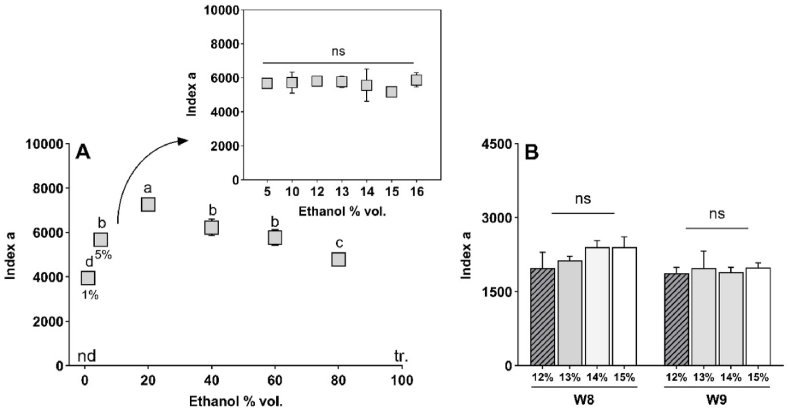
Effect of ethanol level on *index a* of log-normal curve, fitting the formation kinetic of 1-HER in model solution (MW, **A**) and wines (**B**). nd: no detection, tr.: trace. (n = 3, Tukey test, p < 0.05). In **B**, ethanol was spiked in two different wines: W8 is a base wine with initial 11 % vol., W9 with initial 12 % vol. Lowercase letters indicate differences between ethanol spiking levels, ns: no significant difference (n = 3, Tukey test, p < 0.05).

Finally, we examined the impact of ethanol on the formation of 1-HER radical in white wine and model solution, a topic that has not yet been investigated, in order to validate the results obtained in the cluster analysis (Table S4). To do so, preliminary tests were carried out on synthetic solutions covering the 0–100 % vol. range (Fig. 7A and S4). Under our experimental conditions, no signal was detected in the absence of ethanol, which confirms previous observations (Nikolantonaki et al., 2019a).

The evolution of *index a* in the range of 0–20 % vol. is indicative of an increase in 1-HER formation, reaching its maximum (a = 7258) at 20 % vol. Subsequently, it decreased until 100 % vol where negligible 1-HER signals (<5 μM) were detected. In addition, we found that from 5 to 16 % vol., the amount of ethanol did not significantly modify the level of *index a* ($\bar{a}$ = 5762) (Fig. 7A) nor the overall 1-HER formation kinetic.

We evaluated the impact of ethanol addition on the formation of 1-HER in three commercial wines (from 12 to 15 % vol.). The results (Fig. 7B) showed that ethanol had no significant effect on *index a*, confirming the observations made in the model wine solution. This finding shows that the link between *index a* and ethanol level (Fig. S4) is not direct, and is likely to be impacted by the maturity level of the grapes, for example.

### 3.7 Interpretation of the *index a* of the log-normal model

Among the four parameters of the log-normal model describing the formation kinetic of 1-HER, *index a* was the most informative. One of the challenges in modelling chemical reactions is to interpret the natural phenomenon of aging as closely as possible, i.e., to provide an explanation for dimensionless numbers such as *index a*, accounting for the overall trend of the radical formation kinetic. Regarding the trends observed in wines and model solution (12 % vol.) in Fig. 7, a striking finding is the large difference between $\bar{a}$_wine_ = 2094 in the tested wines and $\bar{a}$_ms_ = 5809 in model wines, respectively.

This difference may be explained by the dynamics of the Fenton system. In the model solution, the lack of efficient 1-HER and H_2_O_2_ scavengers sustains Fenton-driven oxidation, leading to prolonged 1-HER formation with slower decay and consequently higher *index a* values. In contrast, white wines exhibit rapid initial 1-HER production due to pro-oxidants, but antioxidants quickly inhibit its production, resulting in a sharper yet transient kinetic profile consistent with lower *index a* values.

## 4 Conclusion

During bottle aging, oxygen ingress generates hydrogen peroxide, which is largely scavenged by free bisulfites. After the bisulfites are depleted, Fenton-type reactions may occur, whereas 1-HER is the main free radical observed in this process. The present findings demonstrate the ability of Spin Trap/EPR spectroscopy to detect unstable and reactive radicals, thereby allowing evaluation of the potential for 1-HER to be formed in wines following the removal of free bisulfites. We optimized the Spin Trap/EPR technique to model the overall kinetic curve of 1-HER formation/degradation through a log-normal function. The first results obtained with several Chardonnay wines from Burgundy show the ability of this method to separate wines according to their 1-HER formation kinetics. Notably, wines with different radical-kinetic profiles can exhibit different levels of major phenolic compounds. Across the 0–80 % vol. ethanol range, ethanol modulated the kinetics in a complex manner. Overall, ethanol and phenolics were not the primary explanatory factors in white wine conditions, so other compounds with a strong influence on radical formation kinetics might be involved. Of course, this approach could be applied to other varieties, with these observations extending to other wines.

## CRediT authorship contribution statement

**Pei Han:** Writing original draft, Methodology, Formal analysis, Data curation, Conceptualization, Investigation, Software. **Alexandre Pons:** Conceptualization, Methodology, Writing review & editing, Resources, Project administration, Funding acquisition, Supervision.

## Declaration of competing interest

The authors declare no conflict of interest.
