# Supplementary material for: Electron paramagnetic resonance (EPR) spin trapping applied to Chardonnay wines: impact of phenolic content and ethanol
Source: Curr Res Food Sci. 2025 Dec 11;12:101276. doi: 10.1016/j.crfs.2025.101276 (PMC12794251; doi:10.1016/j.crfs.2025.101276)
Supplement: Multimedia component 1 [file mmc1.docx]

**Electron paramagnetic resonance (EPR) spin trapping applied to Chardonnay wines: impact of phenolic content and ethanol**

Pei HAN ^a, b^, Alexandre PONS ^a, b, c *^

*^a^ Univ. Bordeaux, Bordeaux INP, INRAE, OENO, UMR 1366, ISVV, F-33140 Villenave d’Ornon, France*

*^b^ Bordeaux Sciences Agro, Bordeaux INP, INRAE, OENO, UMR 1366, ISVV, F-33170 Gradignan, France*

*^c^ Seguin Moreau France, Z.I. Merpins, BP 94, 16103 Cognac, France*

** Corresponding author*

E-mail address:

pei.han@u-bordeaux.fr, ORCiD: 0009-0002-7459-3374

[alexandre.pons@u-bordeaux.fr](mailto:alexandre.pons@u-bordeaux.fr), ORCiD: 0000-0002-0345-8186

# Supplementary material


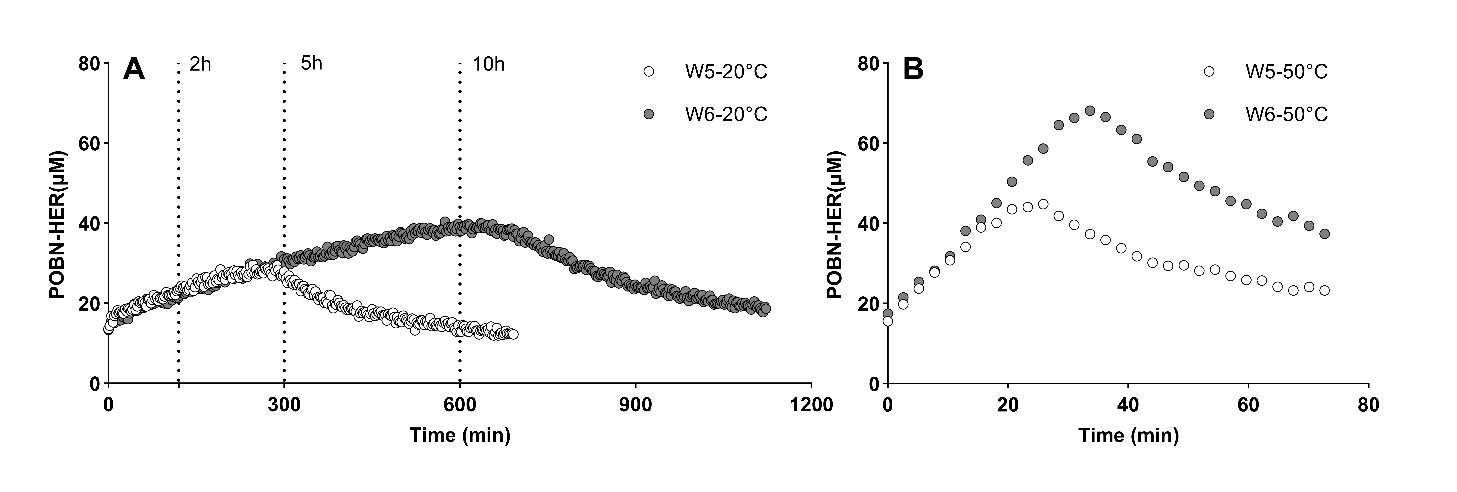


**Fig. S1.** Preliminary tests to compadres the formation kinetic of POBN-HER at room temperature (**A**) and at 50°C (**B**).

**Table S1.** Optimization of cavity temperature test and impact on conventional EPR parameters

|  | **T_max_** ^a^ | | | | | | **[max]** ^b^ | | | | |
| --- | --- | --- | --- | --- | --- | --- | --- | --- | --- | --- | --- |
| **Wines** | **20°C** | **30°C** | **40°C** | **50°C** |  |  | | **20°C** | **30°C** | **40°C** | **50°C** |
| W1 | 450.1 | 191.3 | 96.1 | 51.9 |  |  | | 29.75 | 43.80 | 67.5 | 72.54 |
| W2 | 429.8 | 160.8 | 80.5 | 38.9 |  |  | | 36.81 | 51.15 | 67.92 | 75.57 |
| W3 | 219.2 | 130.0 | 54.5 | 31.1 |  |  | | 34.77 | 40.41 | 40.14 | 49.38 |
| W4 | 272.5 | 160.8 | 70.1 | 38.9 |  |  | | 32.55 | 39.42 | 36.69 | 43.35 |
| W5 | 247.1 | 119.6 | 54.5 | 25.9 |  |  | | 29.54 | 33.78 | 44.07 | 44.79 |
| W6 | 574.5 | 163.4 | 64.8 | 33.7 |  |  | | 40.38 | 54.21 | 59.43 | 68.07 |

^a^ Time to reach maximum (min). ^b^ Maximum concentration (µM).

| **Parameters** | **Repeatability** ^a^ | | |  | | **Reproducibility** ^b^ |  |
| --- | --- | --- | --- | --- | --- | --- | --- |
|  | **RSD** | **SD ^*^** |  | | **RSD** | | |
| T_max_ ^c^ | 3% | 1% |  | | 8% | | |
| [max] ^d^ | 3% | 2% |  | | 5% | | |
| AUC ^e^ | 5% | 1% |  | | 4% | | |
| Index a | 6% | 1% |  | | 4% | | |
| Index b | 2% | 1% |  | | 3% | | |
| Index x_0_ | 4% | 1% |  | | 4% | | |
| Index y_0_ | 5% | 2% |  | | 6% | | |
| R^2 f^ | 0.972 | 0.5% |  | | 0.965 | | |
| ^a^ Three wines W7, W11 and W12 were used to validate repeatability (n = 9 for each wine). ^b^ wine W7 was used to test reproducibility (n = 6). ^c^ Time required to reach maximum concentration. ^d^ Maximum concentration of POBN-1HER during measured period (height of the curve). ^e^ Area under the curve. **^*^** Standard deviation. ^f^ Goodness of fit (R^2^) by means of log-normal function on all the tests. | | | | | | | |

**Table S2.** Validation of method by different EPR parameters of fitting curve


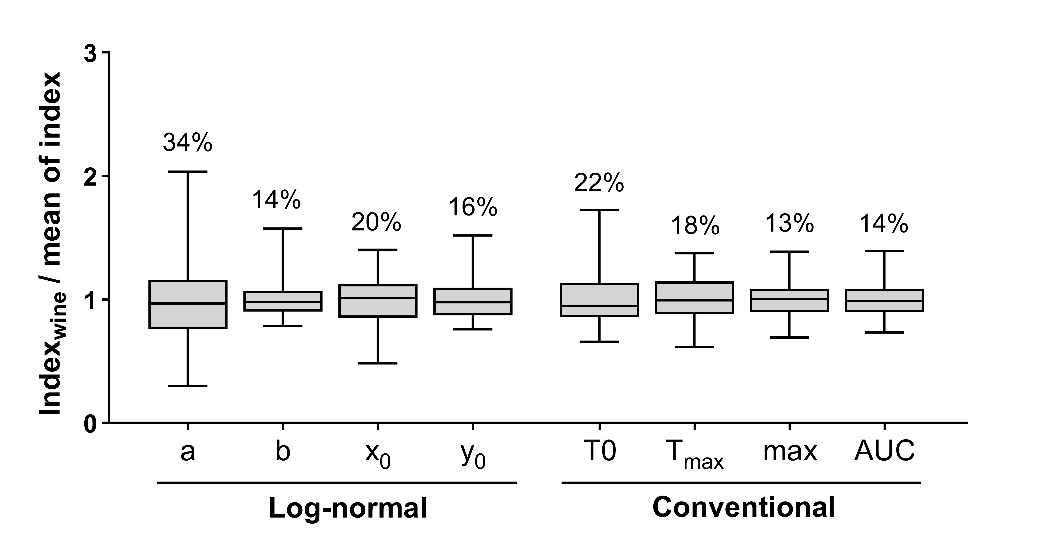


**Fig. S2.** Comparison of discriminant capacity of principal EPR indexes (log-normal and conventional parameters) based on EPR database of 69 Chardonnay wines. Index_wines_ / mean: EPR index of individual sample divided by mean index of 69 wines. Upper values on box plot represents RSD of each index for 69 tested wines.

**Table S3.** Enological analysis and information on wines

| **Wines ^+^** | **Origin*** | **Vintage** | **OD_420_ ^a^** | **Alc. ^b^** | **TA ^c^** | **pH** | **Tar ^d^** |
| --- | --- | --- | --- | --- | --- | --- | --- |
| C1 | CB | 2022 | 0.14 | 12.9 | 4.3 | 3.3 | 3.5 |
| C2 | CB | 2022 | 0.14 | 12.6 | 3.8 | 3.3 | 3.7 |
| C3 | Chablis | 2022 | 0.15 | 13.3 | 3.5 | 3.4 | 3.7 |
| C4 | Chablis | 2022 | 0.15 | 13.2 | 2.6 | 3.5 | 2.7 |
| C5 | Chablis | 2022 | 0.20 | 12.9 | 3.7 | 3.4 | 3.7 |
| C6 | CB | 2022 | 0.14 | 13.2 | 4.3 | 3.3 | 4.5 |
| C7 | CB | 2022 | 0.15 | 13.2 | 3.7 | 3.3 | 3.2 |
| C8 | CB | 2022 | 0.13 | 13.4 | 3.2 | 3.4 | 3.1 |
| C9 | CB | 2022 | 0.18 | 13.7 | 3.8 | 3.4 | 3.3 |
| C10 | CB | 2022 | 0.18 | 13.6 | 3.6 | 3.5 | 3.3 |
| C11 | CB | 2020 | 0.13 | 12.9 | 4.1 | 3.3 | 3.6 |
| C12 | CB | 2020 | 0.13 | 13.0 | 3.9 | 3.3 | 3.4 |
| C13 | Mâcon | 2020 | 0.14 | 12.9 | 3.0 | 3.5 | 3.2 |
| C14 | Mâcon | 2020 | 0.12 | 12.7 | 3.9 | 3.3 | 3.1 |
| C15 | CC | 2009 | 0.18 | 12.8 | 3.6 | 3.3 | 2.8 |
| C16 | Mâcon | 2020 | 0.11 | 12.9 | 3.0 | 3.5 | 3.2 |
| C17 | Chablis | 2018 | 0.09 | 12.4 | 3.2 | 3.3 | 3.0 |
| C18 | Chablis | 2019 | 0.10 | 13.3 | 3.6 | 3.3 | 4.0 |
| C19 | CB | 2020 | 0.12 | 13.5 | 4.5 | 3.3 | 2.9 |
| C20 | CB | 2019 | 0.21 | 14.0 | 3.5 | 3.4 | 3.6 |
| C21 | Mâcon | 2019 | 0.02 | 13.7 | 4.5 | 3.3 | 3.6 |
| C22 | CB | 2019 | 0.13 | 13.3 | 5.0 | 3.2 | 3.9 |
| C23 | Mâcon | 2019 | 0.13 | 13.3 | 3.7 | 3.3 | 3.8 |
| C24 | Chablis | 2019 | 0.12 | 13.3 | 3.9 | 3.4 | 2.7 |
| C25 | Mâcon | 2019 | 0.11 | 13.6 | 3.6 | 3.4 | 3.4 |
| C26 | CB | 2019 | 0.11 | 13.2 | 4.3 | 3.3 | 3.4 |
| C27 | CB | 2020 | 0.11 | 13.1 | 3.7 | 3.4 | 4.0 |
| C28 | BB | 2020 | 0.08 | 12.6 | 4.4 | 3.1 | 4.1 |
| C29 | CB | 2020 | 0.07 | 13.4 | 4.1 | 3.3 | 4.3 |
| C30 | Mâcon | 2020 | 0.12 | 13.1 | 4.1 | 3.3 | 3.9 |
| C31 | CB | 2010 | 0.27 | 13.6 | 4.4 | 3.3 | 3.2 |
| C32 | CB | 2010 | 0.11 | 13.7 | 4.3 | 3.3 | 3.1 |
| C33 | CB | 2016 | 0.10 | 13.4 | 3.8 | 3.3 | 3.7 |
| C34 | CB | 2016 | 0.05 | 13.7 | 3.9 | 3.3 | 3.7 |
| C35 | CB | 2017 | 0.03 | 13.9 | 4.0 | 3.4 | 3.6 |
| C36 | CB | 2017 | 0.10 | 13.9 | 4.0 | 3.4 | 3.5 |
| C37 | Chablis | 2019 | 0.14 | 12.8 | 3.7 | 3.3 | 3.8 |
| C38 | CN | 2022 | 0.15 | 13.1 | 3.4 | 3.3 | 2.7 |
| C39 | CB | 2022 | 0.14 | 12.3 | 3.6 | 3.4 | 3.0 |
| C40 | Mâcon | 2022 | 0.13 | 13.1 | 3.6 | 3.3 | 2.4 |
| C41 | CB | 2019 | 0.18 | 12.8 | 3.8 | 3.2 | 3.6 |
| C42 | Chablis | 2022 | 0.19 | 13.0 | 3.5 | 3.3 | 3.1 |
| C43 | Chablis | 2018 | 0.21 | 12.9 | 3.5 | 3.5 | 2.8 |
| C44 | Mâcon | 2001 | 0.35 | 13.1 | 4.1 | 3.4 | 2.4 |
| C45 | Chablis | 2010 | 0.24 | 13.4 | 4.6 | 3.3 | 3.1 |
| C46 | CB | 2012 | 0.23 | 13.2 | 3.4 | 3.5 | 2.8 |
| C47 | Mâcon | 2013 | 0.25 | 12.7 | 3.8 | 3.5 | 2.7 |
| C48 | CC | 2014 | 0.25 | 13.7 | 3.7 | 3.4 | 2.9 |
| C49 | Mâcon | 2014 | 0.30 | 12.5 | 4.5 | 3.3 | 3.2 |
| C50 | Mâcon | 2002 | 0.29 | 13.2 | 4.0 | 3.3 | 3.2 |
| C51 | Mâcon | 2013 | 0.20 | 12.9 | 3.5 | 3.5 | 2.8 |
| C52 | Mâcon | 1999 | 0.32 | 13.9 | 3.5 | 3.3 | 2.3 |
| C53 | Chablis | 2001 | 0.30 | 13.3 | 4.9 | 3.1 | 3.9 |
| C54 | CB | 1997 | 0.43 | 13.6 | 4.3 | 3.3 | 3.3 |
| C55 | CB | 2001 | 0.37 | 12.6 | 4.1 | 3.4 | 2.8 |
| C56 | CB | 1998 | 0.40 | 14.2 | 3.8 | 3.3 | 3.0 |
| C57 | CB | 2010 | 0.31 | 13.1 | 5.0 | 3.2 | 3.1 |
| C58 | CB | 2012 | 0.21 | 13.5 | 3.3 | 3.4 | 2.6 |
| C59 | Chablis | 2012 | 0.29 | 13.0 | 3.5 | 3.4 | 3.0 |
| C60 | CB | 2010 | 0.40 | 13.0 | 3.7 | 3.5 | 2.6 |
| C61 | CB | 2010 | 0.40 | 13.5 | 4.6 | 3.2 | 3.4 |
| C62 | CB | 2000 | 0.08 | 13.8 | 4.0 | 3.3 | 3.5 |
| C63 | CB | 2014 | 0.14 | 14.2 | 3.8 | 3.4 | 3.1 |
| C64 | CB | 2000 | 0.17 | 13.8 | 4.0 | 3.3 | 3.5 |
| C65 | CB | 2014 | 0.35 | 14.2 | 3.8 | 3.4 | 3.1 |
| C66 | CB | 2021 | 0.01 | 12.3 | 4.0 | 3.4 | 3.0 |
| C67 | CB | 2021 | 0.02 | 11.9 | 4.0 | 3.4 | 3.1 |
| C68 | CB | 2022 | 0.01 | 13.4 | 4.3 | 3.3 | 3.6 |
| C69 | CB | 2022 | 0.01 | 13.1 | 4.1 | 3.4 | 2.8 |
| ^a^ Optical density at 420 nm (OD_420_). ^b^ Ethanol (Alc. % vol.). ^c^ Total acidity (H_2_SO_4_ g/L). ^d^ Lactic acid (g/L). ^d^ Tartaric acid (g/L). ^+^ Chardonnay wines (C). ^*^ Origin of wine samples from Côte de Beaune (CB), Côte chalonnaise (CC) and Côtes de Nuits (CN). | | | | | | | |


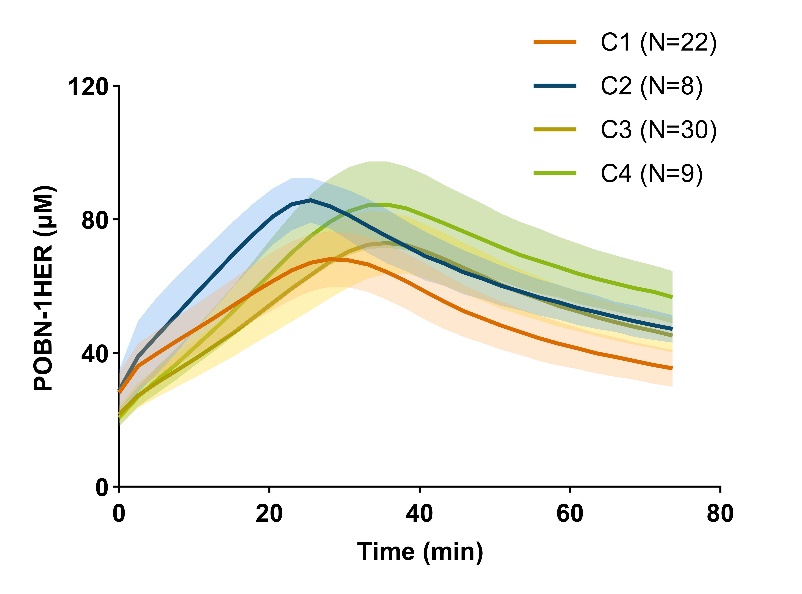


**Fig. S3.** Kinetic curves of 1-HER of 69 wines from four groups. classified by k-means clustering based on their kinetic parameters (log-normal indexes).

**Table S4**. Enological analysis of Chardonnay wines from different clusters categorized by radical kinetic parameters

| Parameters | C1 | C2 | C3 | C4 | p-values ^*^ |
| --- | --- | --- | --- | --- | --- |
| OD_420_ ^a^ | 0.18±0.13 | 0.14±0.03 | 0.18±0.10 | 0.17±0.07 | 0.880 |
| Ethanol ^b^ | 13.2±0.6 b | 12.9±0.1a | 13.3±0.4 b | 13.4±0.4 b | **0.034** |
| TA ^c^ | 4.0±0.4 | 3.6±0.5 | 4.0±0.5 | 3.6±0.5 | 0.121 |
| pH | 3.4±0.1 | 3.4±0.1 | 3.3±0.1 | 3.4±0.1 | 0.232 |
| Tar ^d^ | 3.1±0.4 | 3.2±0.3 | 3.4±0.5 | 3.4±0.4 | 0.146 |
| The enological parameters are expressed as the mean ± standard deviation (SD). Four clusters of Chardonnay wines (C1, C2, C3 and C4) were categorized based on their EPR indexes. ^a^ Optical density at 420 nm (OD_420_). ^b^ Ethanol (Alc. % vol.). ^c^ Total acidity (TA, H_2_SO_4_ g/L). ^d^ Tartaric acid (Tar, g/L). ^*^ P-values of Kruskal-Wallis test, different letters indicated to values correspond to significant differences within clusters (Dunn post-hoc test, p < 0.05). | | | | | |

**Table S5.** Parameter for phenolic compounds analysis.

| TR ^a^ | Compounds | λ _Ex_ ^b^ | λ _Ex_ ^c^ | Standard range | Regression Coeff (R²) |
| --- | --- | --- | --- | --- | --- |
| 5.7 | Gallic acid | 280 | 360 | y = 2001.9x + 351.65 | 0.9974 |
| 8.3 | Caftaric acid | 320 | 490 | y = 420.06x - 127.08 | 0.9982 |
| 12.2 | Hydroxytyrosol | 280 | 320 | y = 89318x - 12732 | 0.9971 |
| 17.7 | Tyrosol | 280 | 320 | y = 357069x + 14381 | 0.9997 |
| 20.4 | Catechin | 280 | 320 | y = 224011x - 15517 | 0.9992 |
| 21.5 | Vanillic acid | 280 | 360 | y = 31198x + 2421 | 0.9967 |
| 23.3 | Caffeic acid | 330 | 470 | y = 1011.3x - 647.43 | 0.9902 |
| 28.7 | Epicatechin | 280 | 320 | y = 193746x - 7790.4 | 0.9996 |
| 33.5 | *P*-coumaric acid | 320 | 440 | y = 2611.4x + 1754.8 | 0.9958 |
| 35.8 | Ferulic acid | 330 | 470 | y = 9289.8x - 5.6639 | 0.9953 |
| 36.4 | *Trans*-resveratrol | 330 | 390 | y = 74689x - 1145.6 | 0.9999 |

^a^ TR: retention tine (min.). ^b^ Excitation wavelengths (λ. nm). ^c^ Emission wavelengths (λ. nm). ^d^ Sum concentration of assayed phenolic compounds.

**Table S6.** Quantification of main phenolic compounds in Chardonnay wines from different clusters categorized by EPR indexes

| Compounds | C1^*^  (N=22) | C2  (N=8) | C3  (N=30) | C4  (N=9) | P-values ^+^ |
| --- | --- | --- | --- | --- | --- |
| Gallic acid | 5.9±3.4 | 10.9±15.1 | 5±4 | 7.7±3.6 | 0.180 |
| Caftaric acid | 49.3±28.9 b | 36.4±14.6 ab | 40.6±24.5 b | 17.7±19.6 a | **0.012** |
| Hydroxytyrosol | 3.9±1.7 b | 2.6±0.9 ab | 3.2±1.4 b | 2.5±1.9 a | **0.016** |
| Tyrosol | 16.5±6.8 | 18.3±5.8 | 18.2±6.7 | 24.1±7.1 | 0.060 |
| Catechin | 2.1±3.5 | 3.3±3.3 | 1.1±0.9 | 1.1±1.1 | 0.315 |
| Vanillic acid | 2±4.5 | 0.7±0.9 | 0.7±0.6 | 2.3±6 | 0.233 |
| Caffeic acid | 8.3±17.1 | 3.5±1.8 | 3.1±1.9 | 2.3±0.6 | 0.440 |
| Epicatechin | 3.1±3.6 b | 6.0±8.0 b | 1.6±1.6 ab | 1.0±1.0 a | **0.043** |
| *P*-coumaric acid | 0.7±0.7 b | 1.6±1.5 b | 0.8±0.7 b | 0.2±0.6 a | **0.010** |
| Ferulic acid | 1.2±1.2 | 1.0±0.7 | 1.2±0.9 | 1.4±0.9 | 0.767 |
| *Trans*-resveratrol | 0.2±0.2 a | 0.1±0.1 ab | 0.1±0.1 b | 0.1±0.0 ab | **0.037** |
| Flavan-3-ols ^a^ | 5.2±6.9 | 9.3±10.8 | 2.7±2.4 | 2.1±2.0 | 0.130 |
| Phenolic acid ^b^ | 67.4±31.2 c | 54.1±22.7 bc | 51.5±25.6 b | 31.5±20.1 a | **0.003** |
| Others ^c^ | 20.5±7.0 | 21.1±5.5 | 21.4±6.3 | 26.6±6.7 | 0.149 |
| Sum ^d^ | 93.2±34.2 c | 84.5±29.1 bc | 75.6±27.4 ab | 60.2±22.1 a | **0.008** |

The concentration of phenolic compounds is expressed as the mean ± standard deviation (SD) in mg/L. ^*^ Four clusters of Chardonnay wines (C1, C2, C3 and C4) were categorized using k-means clustering based on their EPR kinetic indexes. ^a^ Sum concentration of catechin and epicatechin. ^b^ Sum concentration of gallic acid, vanillic acid, caffeic acid, *p*-coumaric acid, ferulic acid and caftaric acid. ^c^ Sum concentration of tyrosol, hydroxytyrosol and *trans*-resveratrol. ^d^ Sum concentration of assayed phenolic compounds. ^+^ P-values of Kruskal-Wallis test. Different lowercase letters indicate significant differences between four clusters (Kruskal-Wallis, Dunn post-hoc test, p < 0.05).

**Table S7.** Spearmen rank correlation coefficient analysis between EPR kinetic parameters and phenolic compounds.

| **Variables** | **Index a** | **Index b** | **Index x_0_** | **Index y_0_** |
| --- | --- | --- | --- | --- |
| Index a | **1** | **0.469** | **0.863** | **-0.449** |
| Index b | **0.469** | **1** | **0.323** | -0.190 |
| Index x_0_ | **0.863** | **0.323** | **1** | **-0.656** |
| Index y_0_ | **-0.449** | -0.190 | **-0.656** | **1** |
| Gallic acid | 0.037 | 0.215 | 0.159 | -0.209 |
| Caftaric acid | **-0.289** | **-0.441** | **-0.246** | 0.216 |
| Hydroxytyrosol | **-0.241** | **-0.435** | -0.219 | 0.125 |
| Tyrosol | 0.226 | **0.316** | 0.169 | -0.166 |
| Catechin | **-0.241** | 0.106 | -0.192 | 0.046 |
| Vanillic acid | -0.074 | -0.108 | -0.117 | 0.087 |
| Caffeic acid | -0.125 | 0.031 | -0.230 | 0.079 |
| Epicatechin | **-0.381** | -0.017 | **-0.330** | 0.166 |
| *P*-coumaric acid | -0.079 | -0.100 | -0.122 | 0.059 |
| Ferulic acid | 0.097 | 0.235 | 0.085 | -0.187 |
| *Trans*-resveratrol | **-0.311** | 0.007 | -0.151 | 0.034 |
| Sum ^a^ | **-0.357** | **-0.263** | **-0.332** | **0.274** |
| Sum flavan-3-ols ^b^ | **-0.322** | 0.018 | **-0.275** | 0.127 |
| Sum phenolic acid | **-0.366** | **-0.372** | **-0.322** | **0.280** |
| Sum others ^c^ | 0.162 | 0.215 | 0.120 | -0.116 |
| Sum benzoic acid ^d^ | 0.056 | 0.191 | 0.141 | -0.104 |
| Sum cinnamic acid and derivative ^e^ | **-0.361** | **-0.398** | **-0.332** | **0.257** |
| Values in bold are different from 0 with a p value < 0.05 (Spearman test). ^a^ Sum concentration of all assayed phenolic compounds. ^b^ Sum concentration of catehin and epicatechin. ^c^ Sum concentration of tyrosol. hydroxytyrosol and *trans*-resveratrol. ^d^ Sum concentration of gallic acid and vanillic acid. ^e^ Sum concentration of caffeic acid, *p*-coumaric acid, ferulic acid and caftaric acid. | | | | |


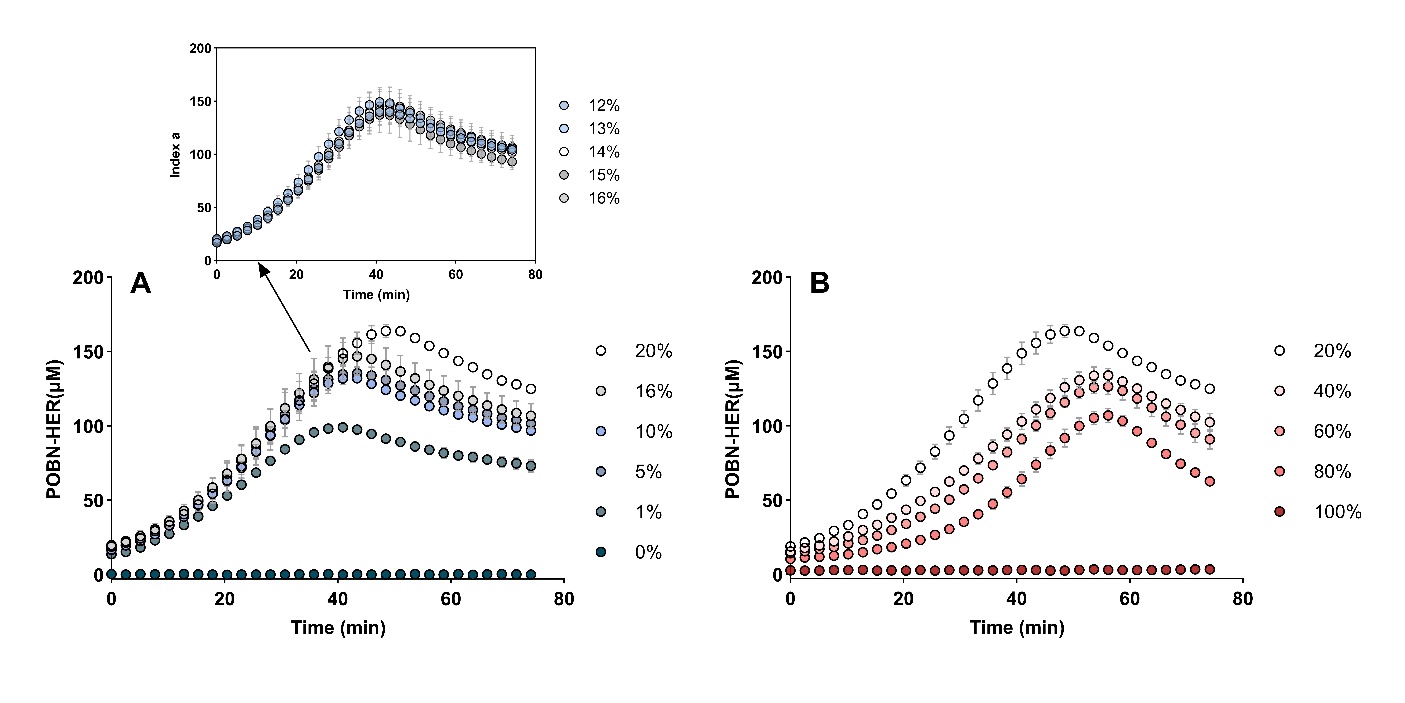


**Fig. S4.** Effect of ethanol content (**A**. range 0 to 20 % vol. **B**. range 20 to 100 % vol.) on formation of 1-HER in model solution (n = 3).
